# Polarized and Evanescent Guided Wave Surface-Enhanced Raman Spectroscopy of Ligand Interactions on a Plasmonic Nanoparticle Optical Chemical Bench

**DOI:** 10.3390/bios14090409

**Published:** 2024-08-23

**Authors:** Xining Chen, Mark P. Andrews

**Affiliations:** Department of Chemistry, McGill University, 801 Sherbrooke St. West, Montreal, QC H3A 0B8, Canada; xining.chen@mail.mcgill.ca

**Keywords:** surface-enhanced Raman spectroscopy (SERS), evanescent guided-wave SERS, guided-wave Raman spectroscopy, magnetoplasmonic, plasmonic waveguides, optical biosensor, optical chemical bench (OCB), optical chip

## Abstract

This study examined applications of polarized evanescent guided wave surface-enhanced Raman spectroscopy to determine the binding and orientation of small molecules and ligand-modified nanoparticles, and the relevance of this technique to lab-on-a-chip, surface plasmon polariton and other types of field enhancement techniques relevant to Raman biosensing. A simplified tutorial on guided-wave Raman spectroscopy is provided that introduces the notion of plasmonic nanoparticle field enhancements to magnify the otherwise weak TE- and TM-polarized evanescent fields for Raman scattering on a simple plasmonic nanoparticle slab waveguide substrate. The waveguide construct is called an optical chemical bench (OCB) to emphasize its adaptability to different kinds of surface chemistries that can be envisaged to prepare optical biosensors. The OCB forms a complete spectroscopy platform when integrated into a custom-built Raman spectrograph. Plasmonic enhancement of the evanescent field is achieved by attaching porous carpets of Au@Ag core shell nanoparticles to the surface of a multi-mode glass waveguide substrate. We calibrated the OCB by establishing the dependence of SER spectra of adsorbed 4-mercaptopyridine and 4-aminobenzoic acid on the TE/TM polarization state of the evanescent field. We contrasted the OCB construct with more elaborate photonic chip devices that also benefit from enhanced evanescent fields, but without the use of plasmonics. We assemble hierarchies of matter to show that the OCB can resolve the binding of Fe^2+^ ions from water at the nanoscale interface of the OCB by following the changes in the SER spectra of 4MPy as it coordinates the cation. A brief introduction to magnetoplasmonics sets the stage for a study that resolves the 4ABA ligand interface between guest magnetite nanoparticles adsorbed onto host plasmonic Au@Ag nanoparticles bound to the OCB. In some cases, the evanescent wave TM polarization was strongly attenuated, most likely due to damping by inertial charge carriers that favor optical loss for this polarization state in the presence of dense assemblies of plasmonic nanoparticles. The OCB offers an approach that provides vibrational and orientational information for (bio)sensing at interfaces that may supplement the information content of evanescent wave methods that rely on perturbations in the refractive index in the region of the evanescent wave.

## 1. Introduction

The binding and orientation of small molecules on surfaces influence many processes in surface physics, chemistry, and catalysis [1,2]. In biosensing, the detection and orientation of molecules on the surface of sensing platforms can affect the interactions between the sensing molecule and the target analyte. Indeed, the sensitivity and selectivity of the biosensor can be improved by controlling the orientation of the sensing molecule at interfaces [3,4,5]. Platforms, like various kinds optical waveguides, can be adapted as lab-on-a-chip (LoC) constructs to enhance biological and chemical sensing [6,7]. These optical LoCs are predicated on device design concepts imported from the photonics industry. Photonic sensor devices include ring resonators, Mach–Zehnder interferometers, multi-mode interference filters, plasmonic metal films, diffraction gratings, and complex silicon photonic crystal integrated circuits. The primary role of these devices is to detect small perturbations in the refractive index induced by changes in analyte concentration within the evanescent optical field of the surface of the device.

Biosensing also benefits from speciation and molecular orientation information based on data that one can collect using Raman spectroscopy. Here, the inherently weak Raman effect also benefits from the implementation of photonic chip paradigms that enhance the laser power density either localized within the bulk of the scattering medium or at the device surface within an evanescent field when a guided wave is confined as a polarization and mode-selected propagating entity in a guided-wave device format [8,9]. The focus of the present work was on an adaptation for Raman spectroscopy to create a LoC slab waveguide construct that we call an optical chemical bench (OCB).

To lay the groundwork for understanding the polarized guided-wave Raman spectra discussed later in this paper, we give a simplified introduction to guided-wave Raman spectroscopy. More detail can be obtained by referring to the citations in reference [9].

**Figure 1:**
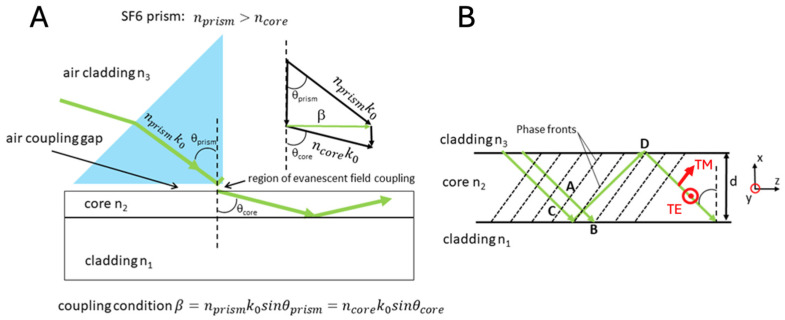
For illustrative purposes, the diagram shows the green arrow ray optics approximation to describe mode and polarization-selected guided wave propagation in a 3-layer dielectric slab waveguide. (**A**) The prism (blue) exhibits a nanoscale coupling gap where the evanescent tail of the ray $n_{prism}k_{0}$ is phase-matched to an eigenmode of the waveguide, meaning that the input optical energy can be transferred from the prism to a wave with a specific nodal structure, field amplitude, and polarization in the film. Note that the refractive index of the core exceeds that of the substrate and the cladding. The inset shows the “vector triangle” that is used to construct and define the propagation constant, β. (**B**) Diagram of the ray optics approximation and coordinate system used to establish the phase fronts (dotted lines) and the orthogonal TE and TM polarizations of the waveguide. While the phase fronts are shown to move between points A and B, they belong to the same plane wave. In the approximation, the ray AB experiences no reflection. Note the longer ray CD. It belongs to the reflected wave. It has experienced two internal reflections while traveling through the phase front at A to the phase front at B. Since all points on the same phase front of the plane wave must be in phase, the optical path length of the ray AB can differ from that of the ray CD only by a multiple of 2*π*.

Briefly, a guided wave can be excited in a medium when the k-vector of incoming light corresponds to an eigenmode of the waveguide. For the 3-layer step index waveguide in Figure 1, the refractive index of the guiding layer $n_{2}$ is a step function and greater than the refractive index $n_{3}$ of the upper air (cladding) and lower $n_{1}$ (substrate) layers. For an optical wave of angular frequency ω and free-space wavelength λ, the media in the three different regions of the waveguide define the following propagation constants:

(1) $$k_{1}=n_{1}\omega /c,\;{\;k}_{2(core)}=n_{2}\omega /c,\;{\;k}_{3}=n_{3}\omega /c\;;\;k=2\pi /\lambda$$

where $k_{2(core)}>k_{1},k_{3}$.

If the angle $\theta_{core}$ is greater than the critical coupling angles for reflection at the top and bottom interfaces, the wave inside the core will be totally reflected at both interfaces and remain trapped by the core. This condition yields guided modes (Equation (2)). As the wave reflects back and forth between the two interfaces, it interferes with itself. A guided mode can exist only when a transverse resonance condition is satisfied such that the repeatedly reflected wave constructively self-interferes. In the core region, the x component of the wavevector is $k_{x}=k_{1}cos\;\theta$ for a ray with an angle of incidence *θ*, while the z component is *β =* $k_{1}$*sin θ*. The longitudinal propagation constant *β* is defined by the vector triangle in Figure 1A, where $\beta^{2}+k_{x}^{2}={\left(n_{2}\omega c\right)}^{2}$. *β* is therefore a real entity that represents the change in phase per unit length along the path travelled by the wave at any instant. Phase shifts $\varphi_{2}$ and $\varphi_{3}$ occur with the internal reflections in the lower and upper interfaces. Because $\varphi_{2}$ and $\varphi_{3}$ are functions of *θ*, the transverse resonance condition for constructive interference in a round-trip transverse passage is

(2) $$2k_{1}dsin\theta +\varphi_{2}\left(\theta \right)+\varphi_{3}\left(\theta \right)=2m\pi$$

where *m* is an integer, *m* = 0, 1, 2,… In a plane wave representation of propagation, the mode number counts nodes in the field.

Two transverse polarization waves exist in the planar structure. The transverse electric field ${TE}_{m}$ points in the y direction and corresponds to perpendicular (s) polarization. The transverse electric field vector lies entirely in the xy plane (i.e., E_z_ = 0) that is transverse to the direction of net travel (the z direction). For the parallel (p) polarization, the electric field is not purely transverse. It has a component along the z direction. This is the transverse magnetic field ${TM}_{m}$. Nevertheless, the magnetic field points in the y direction, so for this polarization, it is entirely transverse (i.e., $H_{z}$ = 0). Overall, the transverse resonance condition results in discrete values of the propagation constant *β_m_* for guided modes, which are identified by the mode number *m*. It is important to note that although the critical angles do not depend on the polarization of the wave, the phase shifts $\varphi_{2}(\theta )$ and $\varphi_{3}(\theta )$, caused by the internal reflection at angle *θ*, depend on the polarization. Thus, TE and TM waves have different solutions for the transverse resonance condition, resulting in different *β_m_* values and different mode characteristics for a given mode number *m*. In general, β_0_ > β_1_ > β_2_ > …

Eigenmode excitation is often achieved with a high refractive index coupling prism or a diffraction grating deposited on the surface of the waveguide (Figure 1A). The incoming beam is rotated through an angle $\theta_{prism}$ to match the resonance condition for waveguiding. In effect, the coupling mechanism solves the eigenvalue Equation (2).

In a dielectric (insulator) medium, guided-wave Raman spectroscopy takes advantage of the fact that one can roughly control the polarization state, TE/TM, of the propagating wave according to the principles described above. For thin enough waveguides and well-defined refractive indices and wavelength, the nodal characteristics and the spatial distribution of the electric field intensity in the waveguide can also be controlled through the mode index. In our experiments, we used 200 μm thick waveguides. At 532 nm and a core index of ~1.460, the slab waveguide supports about 800 TE and 800 TM modes. These cannot be resolved by prism coupling. The waveguide therefore acts more like an attenuated total reflectance (ATR) guided-wave device. For analytes near or bound to the surface of such an ATR guide therefore has only the weak intensity of the exponentially decaying evanescent field to excite Raman scattering. Thus, there is no advantage to using the low refractive index contrast multimode ATR configuration to obtain vibrational and orientational information of the analytes at the thick waveguide surface or in the evanescent field beyond the waveguide. This problem can be circumvented by careful fabrication of thin < 0.1 μm waveguide devices that support only one TE/TM polarization mode [10]. The problem can also be overcome by conferring additional field enhancement capabilities at the evanescent wave/waveguide interface, but only in the very simple context of an inexpensive glass slab waveguide. Our approach using this field enhancement method is described next.

In the present study, we built hierarchical structures (layers) of molecules, nanoparticles, and sensing elements on top of a porous mat of plasmonic nanoparticles “glued” by a molecular adhesive to a silica waveguide–air cladding interface. A polarized multimode TE/TM guided wave is then used to excite localized surface plasmon resonances (LSPRs) at the waveguide–cladding interface. Our approach uses only disposable microscope coverslips and well-known chemical grafting techniques to bind plasmonic nanoparticles to the waveguide surface. In doing so, we circumvent the more complex photolithography and etching processes needed to fabricate dielectric waveguide devices for waveguide-enhanced Raman spectroscopy (WERS) [9,10]. Our method bears some relation to ATR Raman spectroscopy. This latter technique invokes excitations of surface plasmon polaritons (SPPs), usually in the Kretschmann or Otto configuration [11]. In this case, a glass prism is ostensibly used to match the k-vector of the incoming plane-polarized light with the momentum of the SPP, which is excited at the metal–dielectric interface. The SPP propagates parallel to the metal plane. Since the SPP propagates as a wave at the interface, it is very sensitive to changes in the local refractive index, i.e., to the influx or binding of the analyte. In this sense, the SPP circumvents the diffraction limit in optical signal processing [11,12]. Notably, the SPP in the Kretschmann–Otto constructs only propagates over very short (μm) distances owing to the high negative (lossy) part of the dielectric function of the thin noble metal film. In contrast, plasmonic nanoparticles (PNPs) satisfy the coupling condition without need for a prism. Our work also shows that an evanescent guided wave can propagate over distances of centimeters, allowing us to gain the benefit of the Raman scattering from the slit image of the standing wave and its projection onto the slit of the spectrograph.

PNPs continue to figure prominently in detection schemes to enhance biosensor efficacy [13]. For example, gold nanoparticles feature widely in the surface-enhanced Raman spectroscopy (SERS) biosensor literature, in part because of their ease of synthesis, stability, and amenability to chemical conjugation with proteins, antibodies, and other biomarkers [14,15]. In this context, SERS coupled to the electric field response of PNPs continues to mature as a detection and spectroscopic probe technique that is rapid and largely nondestructive, and even sensitive enough for single-molecule detection. A review [16] of the SER effect reveals that low amplitude (10–10^3^) enhancement in SERS relies on near-field enhancements of Raman scattering due to some form of charge transfer electronic coupling between the analyte and the nanoscale features of the enhancing substrate (chemical mechanism); alternatively, an electromagnetic mechanism (EM) accounts for how electromagnetic field excitations of localized surface plasmons enhance the probability of Raman scattering. The LSP response arises from the resonant coupling of the oscillating electric field that drives surface oscillations of nearly free conduction electrons. This coupling produces highly localized and often non-uniform (“hot spot”) surface electric field enhancements [17] with amplification factors up to 10^11^.

Our study also examined the ligand interface between plasmonic and magnetic nanoparticles. The incentive for this comes from the fact that additional enhancement of the SER response can be obtained by fabricating magnetoplasmonic structures. The magnetoplasmonic effect arises from the interplay between plasmonic and magneto-optical (MO) phenomena that can occur in metallic nanostructures. The magneto-optic effect is associated with a change in light intensity or polarization induced by magnetization from an external magnetic field or from a magnetized medium. The phenomenon is usually described in the framework of the magneto-optic Kerr effect (MOKE). More details on the MOKE are available by consulting the review by Singh et al. [18]. Chen et al. reported that γ-Fe_2_O_3_@Au core–shell “magnetoplasmonic” nanoparticles improved the sensitivity of SERS biochips, but the effect they observe is not strictly magnetoplasmonic since the role of the magnetic field in this case was to cause the Fe_2_O_3_@Au nanoparticles to move closer together to create more “hot spots” enhancing Raman scattering further [19]. More commonly, the magnetoplasmonic effect is detected in the Kretschmann configuration [20,21]. Signal modulation is then achieved and enhanced by the MOKE with a magnetic field oriented in the plane of the film (transverse, T) and perpendicular to the propagation plane of incident p-polarized light. The combination of the TMOKE and the plasmonic response is fundamental to the enhanced analytical response of the magneto-optical SPR technique [22].

**Figure 2:**
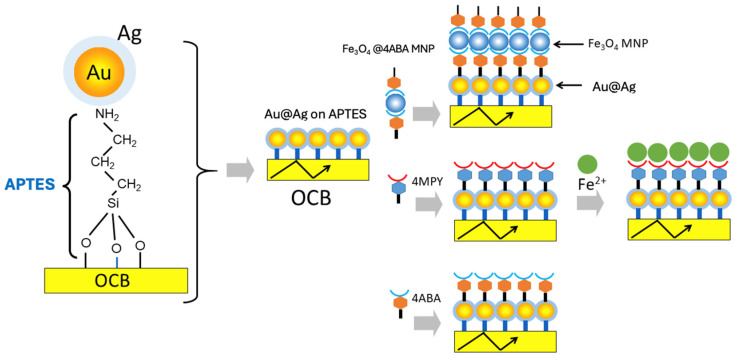
Hierarchies of multilayers of PNPs and molecules on a slab waveguide optical chemical bench (OCB). 3-aminopropyl triethoxysilane (APTES) grafted to the glass waveguide surface is used as a molecular adhesive to bind Au@Ag core–shell nanoparticles. This OCB is further modified by binding magnetite nanoparticles (MNPs) derivatized with 4-aminobenzoic acid (4ABA) to the plasmonic Au@Ag nanoparticles (top center diagram). Alternatively, 4-mercaptopyridine is ligated to the Au@Ag nanoparticles (middle center diagram). The 4MPy ligands can recognize and bind Fe^2+^ or 4-aminobenzoic acid can bind to the Au@Ag nanoparticles (bottom center diagram). The yellow rectangles in the figure indicates waveguides for light propagation. The black arrows within the yellow rectangles indicate propagating light waves.

The discussion thus far raises the question: Can one create a hybrid nanoplasmonic guided-wave structure from a simple glass substrate that avoids complex photonic device microfabrication, circumvents the limitations of the Kretschmann SPP configuration, and utilizes the widely exploited evanescent field, whilst simultaneously co-opting plasmonic enhancement to provide structural and chemical insight into the binding of analytes by coupling a polarized evanescent wave near-field optical response of the SERS effect to a far-field detection scheme? The hybrid structure in Figure 2 was created in response. The structure can be assembled by grafting plasmonic nanoparticles to the surface of a dielectric (glass) waveguide, which admits excitation of polarized evanescent waves in an ATR configuration [23]. This configuration invokes the localized resonant response of PNPs, which are not subject to the constraints imposed by the Kretschmann–Otto configuration. In the present paper, we provide prescriptions for layer-by-layer assembly to make hierarchical nanoplasmonic SERS waveguides on an OCB. In essence, the OCB is a slab waveguide photonic chip—an integrated optics component of a guided-wave Raman spectrograph. The OCB exploits the interaction between the evanescent field at the PNP waveguide surface and the adsorbed analyte, offering low background noise and high specificity [24]. To test the ability of the OCB to sense molecular orientations, nanoparticle binding, ion binding, and hierarchies of assemblies were constructed from the Au@Ag core–shell nanoparticles on the OCB. The adsorbed analytes included 4-aminobenzoic acid (ABA, Figure 2, bottom), 4-mercaptopyridine (MPy, middle), and 4-aminobenzoic acid-functionalized magnetite nanoparticles bound to the Au@Ag nanoparticles (Figure 2, top). The 4-mercaptopyridine construct was used to bind Fe^2+^ (Figure 2, bottom). The sparse nanoplasmonic Au@Ag waveguide cladding and its molecule ad-components were excited with TE- or TM-polarized light from an evanescent wave. The wave was excited by prism coupling. Finite-difference time-domain (FDTD) calculations were carried out to give additional insight into the excitation and interaction of plasmonic nanoparticles near the waveguide surface. Plasmonic waveguide sensors in this format are compatible with the design of current Raman-based planar biosensor chips.

## 2. Materials and Methods

A brief description of the experimental procedure and materials is presented in the following. More details are provided in the Supplementary Information.

### 2.1. Synthesis of Au@Ag Nanoparticles (NPs)

Au@Ag NPs were synthesized using a seed-mediated method adapted from Calagua et al. [25]. A gold seed solution was prepared by dissolving 0.04 g of HAuCl_4_·3H_2_O in 100 mL of Milli-Q water, followed by boiling and the addition of 0.114 g of trisodium citrate dihydrate (Sigma-Aldrich, Oakville, ON, Canada) in 1 mL of Milli-Q water. The mixture was boiled for 15 min and cooled. To grow the gold core, 0.002 g of HAuCl_4_·3H_2_O was dissolved in 20 mL of Milli-Q water, and 20 µL of 1N HCl, 200 µL of gold seed solution, 100 µL of 0.1 M ascorbic acid, and 100 µL of 0.3 M sodium citrate were sequentially added. For the silver shell, 200 µL of 0.1 M AgNO_3_ and 100 µL of 0.1 M ascorbic acid were added dropwise to the gold core suspension. The reaction proceeded for 20 min. The Au@Ag NPs were used immediately.

### 2.2. Preparation of the Optical Chemical Bench (OCB)

OCBs were prepared from 200 μm thick HiQa™ glass coverslips (Sigma-Aldrich), cleaned with soapy water (Sparkleen™, Fisher Scientific, St. Laurent, QC, Canada) and ethanol, then immersed in piranha solution for 1 h (Caution! Piranha solution is strongly acidic and a strong oxidizer as it comprises 3 parts concentrated sulfuric acid and 1 part 30 wt% hydrogen peroxide). After cleaning, the coverslips were functionalized with 0.5 mL of (3-aminopropyl) triethoxysilane (APTES) (99%, Sigma-Aldrich) under anaerobic conditions (Argon) at 70 °C for 1 h. Post-reaction, the waveguides were rinsed with toluene and methanol, dried under vacuum, and annealed at 100 °C for 1 h. The functionalized waveguides were then coated with freshly synthesized Au@Ag NPs to achieve sub-monolayer coverage. Note that we were unable to observe the Raman intensity from the APTES molecular adhesive interface. We expected to see very weak and broad Raman bands in the 3310 and 3360–3380 cm^−1^ regions due to the asymmetric and symmetric NH_2_ stretch modes, respectively. We also expected to see Raman bands in the 2800–3000 cm^−1^ region assignable to the CH stretching modes ν_asym_ CH and ν_symm_ CH of the methylene groups of PNP-bound APTES that might reflect the conformationally ordered or disordered state of hydrocarbon chains. Repeated waveguide SERS experiments did not reveal any signal above the noise that we could reliably conclude belonged to the molecular adhesive layer. The reason for this is likely because the propyl chain and amino groups are poor Raman scatterers, which is also why we selected them so that their contribution to the SER spectra would not interfere with interpretations of the SERS data from our probe molecules.

### 2.3. Binding of 4MPy and 4ABA Molecules

The functionalized OCBs were submerged in 6 × 10⁻^3^ M ethanol solutions of 4-mercaptopyridine (4MPy, Sigma-Aldrich) or 4-aminobenzoic acid (4ABA, Sigma-Aldrich) for 30 min with occasional agitation to ensure uniform binding of the molecules to the Au@Ag-coated surface. We did not quantify the number of 4MPy or 34ABA molecules bound to the OCB.

### 2.4. Fe^2+^ Binding to 4MPy on the OCB

A 0.5 mM Fe^2^⁺ solution was prepared by dissolving 152 mg of FeSO_4_ in deionized water. The 4MPy-functionalized Au@Ag OCBs were immersed in the Fe^2^⁺ solution and agitated lightly. Samples were collected after 0, 8, 20, 35, and 50 min of reaction time.

### 2.5. Binding of 4ABA-Functionalized Magnetite Nanoparticles (MNPs)

Magnetite nanoparticles were synthesized via a coprecipitation method. A total of 1.5 g of FeCl_2_·4H_2_O and 4.1 g of FeCl_3_·6H_2_O were dissolved in 100 mL of deionized water at 70 °C under a nitrogen atmosphere. A 20 mL volume of 5 wt% NH_4_OH was added dropwise until the solution turned black. The MNPs were collected by magnetic separation, washed with deionized water, and functionalized by incubation in a 2 × 10^−2^ M 4ABA solution in ethanol. The surface-modified MNPs were extracted using a magnet and re-dispersed in ethanol. The functionalized MNPs were then deposited on the Au@Ag-coated OCBs.

### 2.6. Characterization Techniques

- X-ray Photoelectron Spectroscopy (XPS): XPS was performed using a Thermo Scientific K-Alpha spectrometer with an Al-Kα X-ray source, operating under a vacuum of <2 × 10⁻^7^ mbar. Spectra for N (1s) and O (1s) were collected for MNPs before and after 4ABA functionalization.
- Integrated Optics (IO) SERS Spectrometry: Polarization-dependent SERS spectra were acquired using a custom-built IO-SERS instrument containing a Triax 550 Raman monochromator (Horiba Scientific, Piscataway, NJ, USA). Details of the custom-built IO-SERS setup is described in SI. A 532 nm CW laser diode was used, and polarized waveguide modes were excited via prism coupling.
- Scanning Electron Microscopy (SEM): High-resolution SEM and EDX were performed using a Helios NanoLab™ 660 instrument (Thermo-Fisher Scientific, St. Laurent, QC, Canada).
- Transmission Electron Microscopy (TEM): Light-field and dark-field TEM, along with EDX, were performed using an FEI Titan Krios 300 kV Cryo-STEM (Thermo-Fisher Scientific, St. Laurent, QC, Canada).
- Simulation of Plasmonic Electric Field: Three-dimensional finite difference time domain (3D FDTD) simulations were conducted using Lumerical Solutions software ((2020 R2), Vancouver, BC, Canada). to model the resonant optical response of the Au@Ag nanoparticles on the waveguide.

### 2.7. Guided-Wave Raman Spectroscopy and Prism Coupling

A custom-fabricated SF6 prism (Precision Optical, Costa Mesa, CA, USA) was used to couple the laser beam into the slab waveguide OCB. The coupling angle required to launch the guided wave was interrogated by varying the rotation angle of the laser beam incident on the prism. This setup enabled precise control over the TE and TM polarization states, essential for the polarization-dependent SERS measurements. Detailed descriptions of the coupling procedure, setup, and theoretical background are provided in the Supplementary Information.

## 3. Results and Discussion

### 3.1. Characterization of OCB Overlayed with Au@Ag Nanoparticles

**Figure 3:**
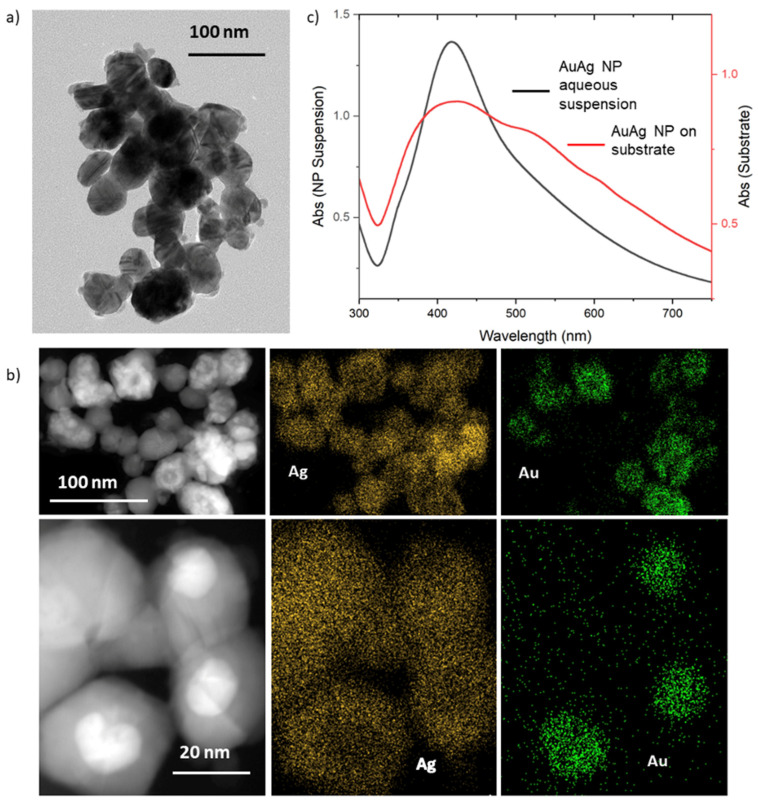
(**a**) Bright-field TEM image of the Au@Ag NPs; (**b**) dark-field TEM images and EDX elemental analysis of Au and Ag components of the Au@Ag and alloy NPs. Gold color indicates Ag, green color indicates Au; (**c**) UV–Vis extinction spectra of Au@Ag NPs suspended in water and after immobilization on a glass substrate.

TEM imaging (Figure 3a) revealed faceted plasmonic NPs with an average size of 49.2 ± 14.4 nm. The light and dark color bands on the NPs may correspond to stacking faults [26]. Due to the similarity in contrast of silver and gold, the two components were difficult to distinguish with bright-field SEM imaging. In contrast, dark-field SEM with energy-dispersive analysis by X-ray (EDX) analysis (Figure 3b) revealed evidence of core–shell particle structures, as well as particles made of homometallic silver or a mix of silver and gold. The nanoparticles containing a mix of silver and gold may be attributed to an Au–Ag alloy, since Au–Ag alloys are known to have more negative formation energy than their core-shell counterparts, indicating that the alloy phase is more stable [27].

Lim et al. found that core–shell Au@Ag NPs have two distinct plasmon absorption bands whose relative intensities depend on the thickness of the shell [28]. The extinction spectrum of the NPs in water (Figure 3c) showed a dominant peak at 424 nm, consistent with a thicker Ag shell. The gold core contributed a weak shoulder to the spectral response, which was visible near 520 nm. Compared to homometallic gold or silver, the core–shell nanoparticles exhibited a broader range of extinction with the extinction intensity extending well into the red that would not normally be observed for isolated Ag NPs. Overall, the spectrum is not that of a mixture of separate Ag and Au NPs, nor is it the result of significant contributions from alloy formation [29,30].

### 3.2. SER Spectra of 4-Mercaptopyridine (4MPy) Binding on Au@Ag NP Surface

**Figure 4:**
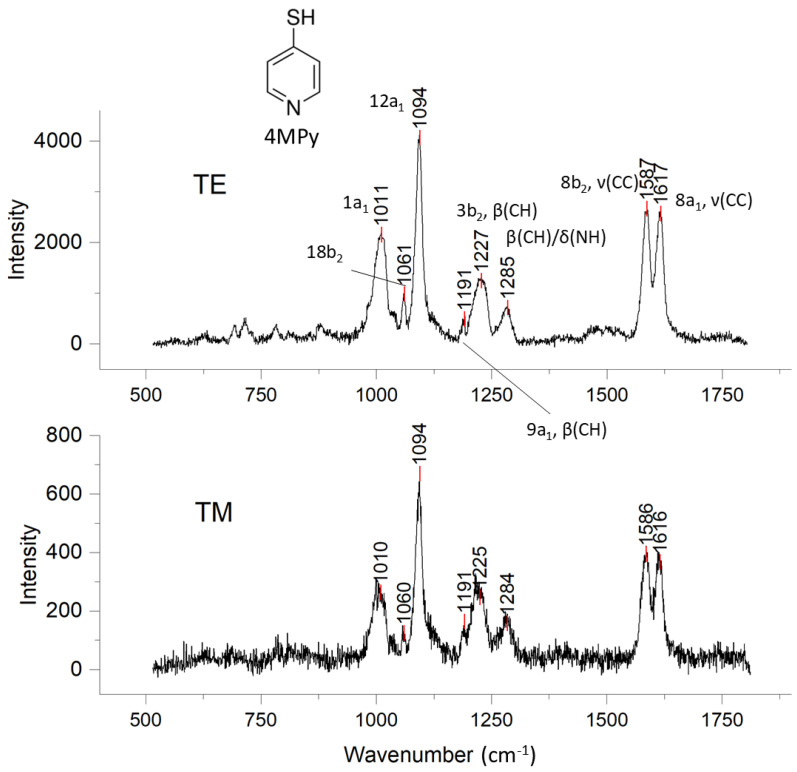
TE and TM SER spectra of 4MPy adsorbed on Au@Ag NPs on an OCB waveguide.

The TE-/TM-polarized evanescent wave SER spectra obtained for 4MPy adsorbed on the Au@Ag NPs of the OCB are shown in Figure 4. The peak assignments for the SER spectra were based on previous studies where the Raman spectrum of 4MPy and its SER responses on different types of silver substrates were examined [23,31,32]. The Raman peaks at 1061, 1191, and 1227 cm^−1^ belong, respectively, to the 18b2 β(CH), 9a1 β(CH), and 3b2 β(CH) in-plane bending modes of the pyridine ring. The peaks at 1587 and 1617 cm^−1^ originate in 8b2 and 8a1 νCC pyridine carbon stretching modes. The absence of a peak for ν(SH) is attributed to the X-sensitive vibrations of 4MPy, which are mixed modes that result from the coupling of the 1a1 ring breathing mode with the vibration of the substituent (sulfur) of pyridine in the 4-position [27]. With this reasoning, the 1a1 ring breathing mode at 1011 cm^−1^ is coupled with the 12a1 species at 1094 cm^−1^, which is the most intense feature in this spectrum region, indicating that the sulfur substituent moves with significant amplitude when bound to Ag/Au.

The Raman evidence suggests that 4MPy coexists as the neutral and the protonated species when bound to Au@Ag on the OCB, but it is not bound to the metal through the nitrogen atom. Protonation of 4MPy is known to weaken and shift the 9a1 β(CH) 1191 cm^−1^ peak to a higher wavenumber in the range between ~1200 and ~1225 cm^−1^ [31,32], making it difficult to distinguish from the 3b2 β(CH) band located at 1227 cm^−1^. The 8a1 νCC band for the non-protonated nitrogen occurs around 1580 cm^−1^ [31,33], while the 8a1 νCC peak at 1617 cm^−1^ is a signature for protonated nitrogen [33,34]. In other words, SERS of the OCB detected both protonated and non-protonated 4MPy. Finally, the peak at 1285 cm^−1^ was assigned to β(CH)/δ(NH) [32], while minor peaks below 900 cm^−1^ were likely from pyridine ring deformation and aromatic CH out-of-plane bending modes [31].

### 3.3. SER Response of 4MPy to TE and TM Evanescent Wave Polarization

Excitation of SERS from 4MPy by TE- or TM-polarized waves did not produce significant differences in the number of peaks nor in the peak positions over the 500 to 3200 cm^−1^ spectral range; however, the Raman counts were uniformly reduced with TM polarization compared to TE polarization. This outcome is consistent with the theoretical predictions of Chen et al. [35], who found that the field enhancement factor for Raman scattering with the TE mode in a polarized plasmonic gold film waveguide was larger than that of the TM mode in the same polarized waveguide. Metal overlayers on dielectric waveguides act as “mode strippers”, preferentially removing TM polarization from mixed TE/TM polarization waves. A metal overlayer on a dielectric waveguide behaves like a high-loss medium with a complex dielectric function $\epsilon (\omega )={\epsilon (\omega )}_{1}-i{\epsilon (\omega )}_{2}$ over the frequency range ω of light [36] because the inertia of charge carriers inside a metal film dominates above the metal plasma frequency $\omega_{p}$. As a result, a metal-clad waveguide has a complex propagation constant k=β−iα(α>0) along the propagation direction. Selective attenuation of the TM polarization is known to depend strongly on the thickness of the buffer (insulator) layer between the metal and the guiding layer [36]. In our case, the buffer layer was roughly the thickness of the APTES ligand interlayer between the Au@Ag and the glass slab waveguide. Thus, it is not surprising that we observed a greater reduction in Raman signals for TM-polarized light. 

### 3.4. Orientation of 4MPy on Au@Ag Nanoparticles on the OCB

Previous studies found that when 4MPy adsorbs onto silver NP and film surfaces, it assumes a roughly vertical orientation [23,31]. The SER data for 4MPy adsorbed on the Au@Ag PNP OCB are consistent with these previous studies. In the 500–1800 cm^−1^ region, the a1 and b2 modes of 4MPy were enhanced compared with the out-of-plane a2 and b1 vibrations (Figure 4). For an upright pyridine orientation, the a1 and b2 modes are normal to the surface. This is consistent with the evidence and interpretations that SER-responsive modes perpendicular to the surface receive the largest enhancement [23,31,37]. In contrast, the out-of-plane a2 and b1 vibrations that are roughly parallel to the surface receive only minor enhancement [23,31]. The Au@Ag PNP SER spectra suggest that 4MPy was bound through the sulfur atom and was predominantly vertically oriented.

### 3.5. Binding of Fe^2+^ to 4MPy

**Figure 5:**
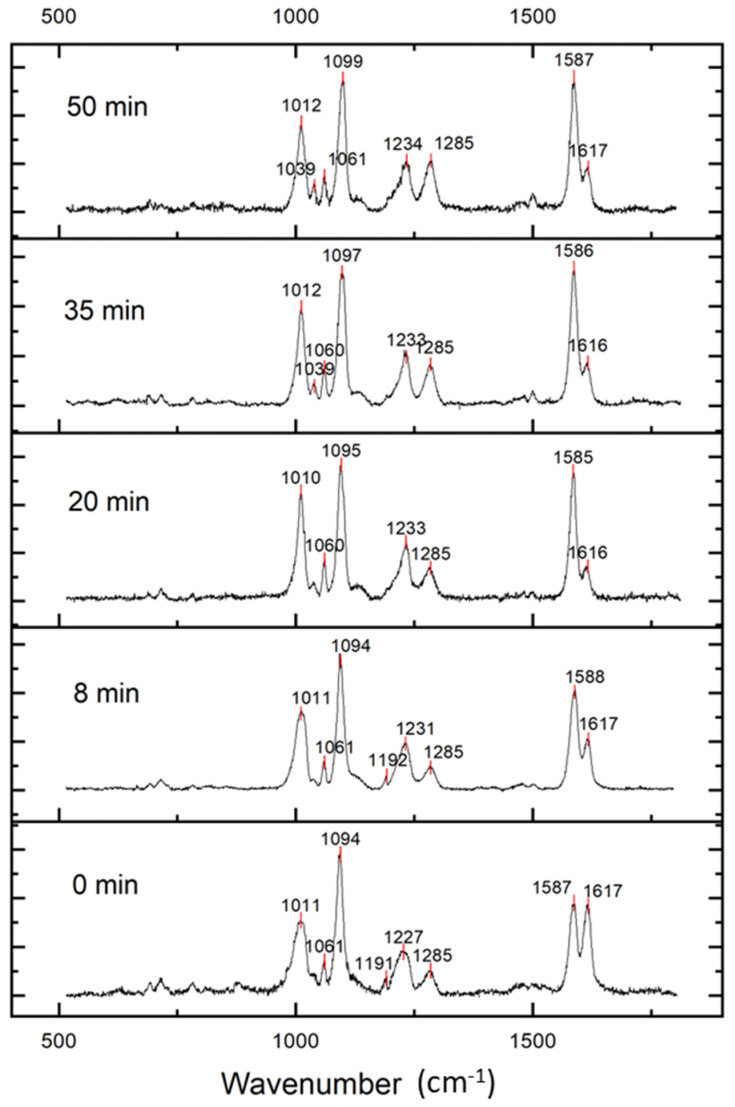
Guided-wave TE-polarized SER spectra of 4MPy molecules adsorbed on the Au@Ag OCB prior to and after different times of exposure to Fe^2+^ solution.

Anticipating our studies of binding magnetite nanoparticles to the Au@Ag OCB, we first examined the capacity of the OCB to detect Fe^2+^ binding since the OCB architecture involves some complexity in terms of the local environment of the 4MPy ad-ligand. We focused on the nitrogen moiety because our studies detailed in Section 3.2 showed that 4MPy exists in both the protonated and deprotonated forms, a fact that was also confirmed elsewhere by XPS studies [38]. For context, we note that nitrogen donor ligands like 4-(pyridin-3-yl)benzoic acid bind to both the ferric and ferrous forms of cytochrome P450 enzymes through the nitrogen atom [39]. Exposure of the OCB to Fe^2+^ from aqueous FeSO_4_ caused some SER bands to increase in intensity relative to others. Figure 5 reveals that exposure of 4MPy to Fe^2+^ ions over time was accompanied by a diminished band intensity at 1617 cm^−1^, associated with the 8a1 ν(C = C) ring mode. In contrast, the β(CH)/δ(NH) (from protonated 4Mpy) and 8b2 β(CH) modes located at the band positions 1285 and 1587 cm^−1^ were enhanced. The increase in the intensity of the 1587 cm^−1^ band and the decrease in the intensity of the 1617 cm^−1^ band indicate the binding of Fe^2+^ in a nitrogen coordination environment that might include coordinated water. Enhancement of the β(CH)/δ(NH) band at 1285 cm^−1^ certainly indicates a change in the pyridine nitrogen coordination environment. On the other hand, Lewis base donation of the deprotonated nitrogen lone pair to Fe^2+^ can be accompanied by delocalization of Fe^2+^ d-orbital electron density into π* acceptor orbitals of the pyridyl ring [37]. The perturbation experienced by backdonation from Fe^2+^ binding might be the cause of the changing intensity of these a1 and b2 modes.

### 3.6. SERS of 4-Aminobenzoic Acid (4ABA) Binding on OCB in Comparison to That of 4MPy

**Figure 6:**
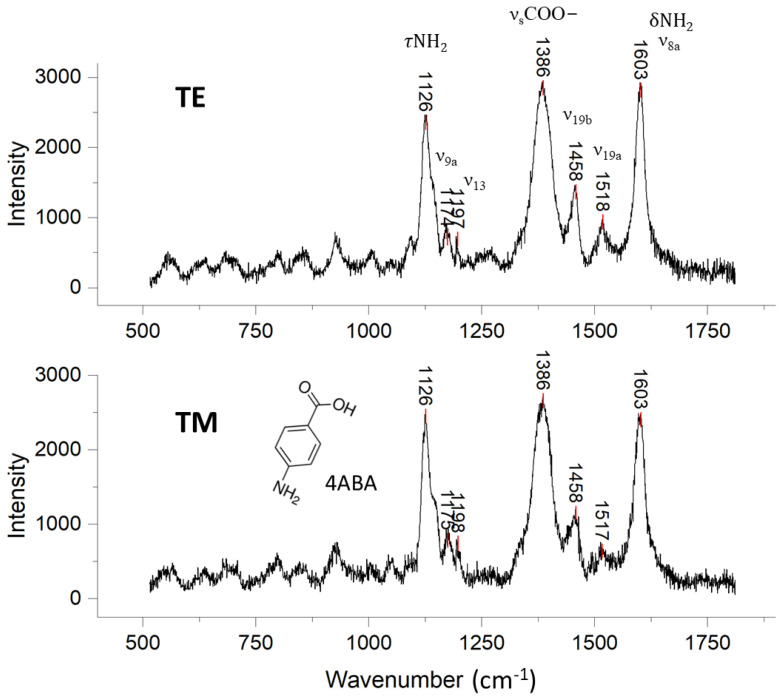
TE and TM SER spectra of 4ABA molecules adsorbed on the Au@Ag OCB.

Figure 6 shows the TETM-polarized SER spectra of 4ABA molecules bound to Au@Ag PNPs on the OCB. The unenhanced Raman spectrum of crystalline 4ABA is provided in Figure S5 for comparison. From the OCB of adsorbed 4ABA, only small differences were observed in Figure 6 between TE and TM SERS intensities and line shapes over the 1000–1750 cm^−1^ range. It is surprising that we did not detect attenuation in the Raman counts for TM polarization, as we did when probing the response of 4MPy (Figure 4). We speculated at this point that the evanescent field might be interrogating regions of the OCB that are very sparse in nanoparticles but still capable of providing a strong SER response.

The most prominent peaks from TE- or TM-polarized wave excitation in the 4ABA SERS occurred at 1126, 1386, and 1603 cm^−1^, attributable to NH_2_ bending (τNH_2_), COO^−^ stretching (νCOO^−^), and NH_2_ deformation (δNH_2_), respectively [40,41]. In the Raman spectrum of crystalline 4ABA (Figure S5), the τNH_2_ mode was weak while the νC-OH mode appeared as a peak of medium strength at 1293 cm^−1^ [42]. The SERS results in Figure 6 show a significant enhancement in the τNH_2_ mode and a blue shift and moderate enhancement of the COO^−^ stretching mode, suggesting that both the amine and carboxylic acid groups of 4ABA are involved in binding to the Au@Ag PNPs, and perhaps that the 4ABA molecule is not upright but inclined or even flat on the PNP surface (see Section 3.7 below).

Elsewhere, studies have indicated that 4ABA may bind to an Ag electrode surface through both the amino and the carboxylic acid groups [43]. In this case, the carboxylic acid moiety was shown to attach via both oxygen atoms to two adjacent Ag atoms in a symmetric μ-bridging fashion on the metal surface [44]. This binding mode was also confirmed for a Au surface [45]. Amino acids have been reported to attach to metal surfaces by donating the nitrogen lone pair to the symmetry and energy-adapted crystal orbitals of the metal, or by accepting the electrons from the metal to the antibonding orbitals of N-H bonds [46]. Based on these above studies, we assigned the strong peak at 1603 cm^−1^ to the overlap of the ν8a 4ABA ring stretching vibration and δNH_2_ deformation. In crystalline 4ABA, the ν8a band exhibited the strongest intensity among all the Raman modes, while the δNH_2_ band exhibited a much weaker intensity and often appeared as a shoulder to the ν8a peak. Like the τNH_2_ mode, the intensity of the δNH_2_ mode was greatly enhanced upon binding to a plasmonic metal surface. Past research has shown that the δNH_2_ band is strongly enhanced due to interactions with a silver surface, surpassing the ν8a band in intensity [41]. Furthermore, we assigned the peaks at 1174, 1197, 1458, and 1518 cm^−1^ to ring stretching modes ν9a, ν13, ν19b, and ν19a, respectively [40]. The dimerization of 4ABA on a metal surface can produce *p*, *p*′-azodibenzoate, which may have been observed in our experiment as a shoulder at 1144 cm^−1^, which overlapped with the ν19b band [43]. Based on the molecular structure of 4ABA and our Raman assignments, both the amino and carboxyl functional groups appear to interact simultaneously with the metal surface, suggesting a horizontal binding orientation on the Au@Ag NP surface.

### 3.7. Caveat on Assignment of Molecule Orientation on Plasmonic Au@Ag PNPs on the OCB

It is helpful to remind the reader that the matter of assigning molecule orientation using the Raman selection rules that have been developed for SERS comes with a caveat. The SERS intensity for a given Raman mode depends on the symmetry of its tensor with respect to the plasmonic surface for a fixed molecular orientation; however, obtaining knowledge of the Raman tensors of different modes is difficult, and the tensors can change on adsorption, as in the case of chemisorption. Moreover, it is difficult to describe local field polarization at the molecular level and how that polarization connects with the Raman enhancement factor. Some advances have been made regarding these problems, especially for the case of a flat metal surface [47], including the research on the (flat) plasmonic waveguide published by Chen et al. [35]. Our qualitative analysis of the orientation of 4MPy and 4ABA presumes that the local field polarization is primarily perpendicular to the nanoparticle surface. Under this assumption, modes having a substantial Raman tensor component normal to the surface will be enhanced more than those whose normal components are weak or forbidden by symmetry. By comparing the SERS enhancement factor of various modes and relating these to the Raman tensors describing the unenhanced, unbound molecule, one can infer an approximate orientation—flat or upright on the surface. Underlying this approach are a number of conditions that need to be met. The requirement that the polarization is perpendicular to the surface is difficult to justify in the case of rough metal surfaces, colloidal suspensions, and most likely, nanoplasmonic particles on the OCB. Furthermore, the enhancement factor for various Raman modes must be distinguishable from other effects, notably the dispersion in the plasmon resonance, which causes the enhancement factor to depend on the Raman shift [48]. Given these challenges, one must resort to qualitative arguments around the SER selection rules to assign molecular orientation. Hence, the arguments for the different orientations of 4MPy and 4ABA remain qualitative.

### 3.8. Exploring the Nanoscale Interface: Detection of 4ABA-Functionalized Magnetic Nanoparticles (MNP) Using Evanescent Waveguide SERS on the OCB

**Figure 7:**
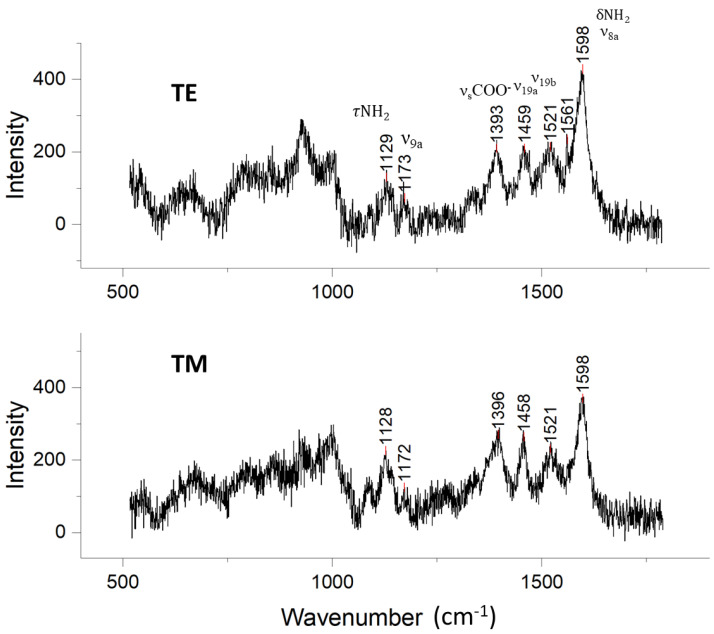
TE and TM SER spectra of 4ABA-functionalized MNPs deposited on Au@Ag PNPs on the OCB.

This section connects with our introductory remarks on magnetoplasmonic enhancement of SERS [18,19]. Here, we used the OCB to probe the nanoscale ligand interface between magnetite nanoparticles and Au@Ag PNPs on the OCB. Our exploration here anticipates future developments in magnetoplasmonic nano-antennas [49]. Refer to Figure 2, top right-hand-side image. We functionalized superparamagnetic magnetite nanoparticles with 4ABA, chelating the MNPs via the 4ABA carboxylic acid group (see Experimental Procedure section in the Supplementary Information). These nanoparticles were then deposited on the OCB, which was pre-decorated with the Au@Ag PNPs. Binding of 4ABA through both the acid and amine functionalities was confirmed by XPS (See SI Figure S4B). We used the predominantly outward pointing amine termini of the MNP@4ABA to bind to the PNP substrate. Raman spectra were gathered by scattering from TE- and TM-polarized guided waves. The SER spectra in Figure 7 show peaks belonging to 4ABA directly bonded to Au@Ag PNPs (compare to Figure 6). Analysis of the peak positions and relative intensities revealed differences between the two types of bound 4ABA (Figure 6 and Figure 7). The τNH2 and νsCOO^−^ bands were blue shifted and reduced in intensity for 4ABA-decorated MNPs on the PNP OCB. This suggests a change in the binding environment. The ν19a mode was also blue shifted, but its relative intensity increased compared to the ν19b and ν9a ring modes. The peak at 1598 cm^−1^ dominated the spectra, and it was red-shifted from its original place at 1603 cm^−1^.

It is also notable that TE or TM guided waves yield slightly different SERS profiles. Compared to their TE counterparts, the τNH_2_, ν_s_COO^−^, and ν19b bands had slightly stronger intensities, while the peak at 1598 cm^−1^ was noticeably weaker. The presence of bands attributable to τNH_2_ and ν_s_COO^−^ indicated that at least some NH_2_ and COO^−^ groups had partially dissociated from the MNP surface and may have bonded with the Au@Ag surface. However, the fact that these two bands were no longer dominant suggested that 4ABA did not assume the same orientation observed when binding to Au@Ag NPs in the absence of the MNPs.

The SERS results from the OCB 4ABA-MNPs varied according to the TE and TM polarization state, unlike 4MPy and 4ABA on Au@Ag NPs alone. Note also that the TE and TM Raman spectral profiles for 45°-angle Raman scattering from crystalline powders (pseudo-orthorhombic unit cell crystallites) are indistinguishable, as shown in Figure S5 [50]. It can therefore be inferred that the 4ABA ligands deposited with the MNPs, now partially bound to the Au@Ag surface, exhibited orientations that were not upright, horizontal, or completely randomized, suggesting a complex environment for the surface ligands.

### 3.9. FDTD Simulation of Electric Fields of Au@Ag NPs Excited by TE/TM Polarized Light

Since the Raman process is proportional to local electric field intensity [16,17], it makes sense that methods to enhance the local field might enhance the Raman response. It is well-known in SERS experiments that when plasmonic nanoparticles aggregate, optical coupling between particles in the electromagnetic field becomes very strong, resulting in a significant increase in the local field near the particle. The particle dimension, state of aggregation, and geometry play a large role in determining the enhancements in plasmon resonance spectroscopy. The incident light around the plasmon resonance peak wavelength is scattered strongly, leading to the SER effect. Regarding the SER effect, it was also established that the coupling between a molecule and the electromagnetic field is enhanced by placing the molecule between two silver nanoparticles [51]. This has the effect of creating “hot spots” that enhance Raman scattering.

**Figure 8:**
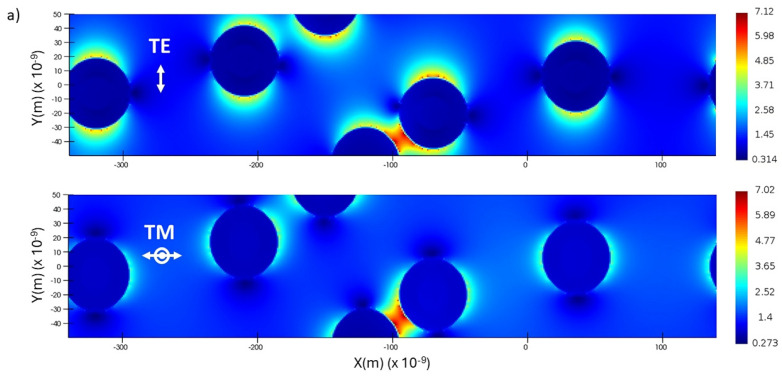

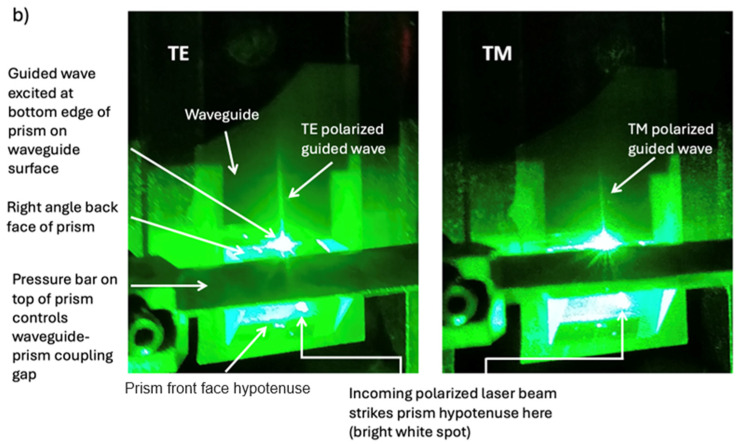
(**a**) FDTD electric field simulations of Au@Ag core–shell particles. The particle dimensions and positions were mapped directly from high-resolution SEM images of the real waveguide (see Figure S3 for details). The color bar on the right indicates the maximum and minimum electric field intensities corresponding to either TE or TM polarization. The vertical axis in each simulation is y, and the horizontal axis is x. The double-headed arrow (TE) indicates oscillations in the plane. The arrow and dot (TM) indicate oscillations orthogonal to the plane of the waveguide. (**b**) Prism coupling assemblies and still images of propagating guided-waves for polarized TE and TM multimode excitation. The pressure bar controls the coupling gap between the bottom of the prism and the waveguide surface. Incoming light incident at the prism hypotenuse face (white spot) is refracted to the bottom back edge of the prism where it excites closely spaced eigenmodes of the waveguide. The 532 nm guided wave is resonant with the red end of the extinction spectrum of the plasmonic nanoparticles (Figure 3a,c). Extinction accounts for the attenuation of the beam as it propagates. Note that the TM wave is more strongly attenuated than the TE wave (see text for details). A visual manifestation of the greater damping of the TM polarization can be seen in (**b**) which shows the shorter propagation distance of the 532 nm guided wave when the polarization is switched from TE to TM.

To investigate the interaction between the TE- and TM-polarized evanescent waves and the plasmonic nanoparticles attached to the OCB surface, FDTD calculations were carried out based on the simplification depicted in Figure S3. Figure 8a shows plots of the electric field intensity around Au@Ag NPs as they are excited by the evanescent field of TE- or TM-polarized light propagating in the waveguide beneath the nanoparticles. Figure S3 explains that the scattering centers were mapped onto the simulation directly from ultra-high-resolution SEM images. Excitation with the TE mode led to a symmetric field intensity concentrated at the north and south poles of the particles and nodes around the equator of particles. TE polarization (Figure 8a) was in the xy plane oriented in the direction of the double-headed white arrow. For TM excitation, the electric field oscillated orthogonally to the xy plane. The field intensity concentrated around the equator of the nanoparticles and had a lower intensity at the surface than that shown for the TE response. Particles isolated far enough apart showed an undistorted dipolar field intensity. For both polarizations, as the interparticle separation decreased, one could observe distortions in the fields. With further decreases in the interparticle distance (bottom center, Figure 8a), one could observe the high concentrations of field intensity that are the hallmark of the “hotspots” that are associated with enhanced SERS [17,52].

TE- and TM-polarized light exhibit different transmission losses as the light propagates in asymmetric, metal-clad, thin-film-clad waveguides [53]. The attenuation experienced by the TM mode was greater than that of the TE mode (Figure 8b) because the TM mode interacts strongly with the metal surface, while the TE mode is repelled by the metal surface [53]. The plasmonic particles on the OCB behaved as a polarization mode filter. Nevertheless, the discontinuous nature of the plasmonic Au@Ag NP overlayer allowed us to propagate polarizations over distances larger than would be capable with a continuous metal film.

## 4. Conclusions

In this study, an integrated optics setup with an OCB was prepared by immobilizing Au@Ag PNPs on a glass substrate using APTES. This system formed a porous nanoplasmonic particle mat that was used to excite the SER of adsorbates by resonant coupling of TE- or TM-polarized waves with the conduction electrons of the surface-bound plasmonic nanoparticles (PNPs). Hierarchies of assemblies of 4MPy, 4ABA, and magnetite nanoparticle adsorbates were examined by polarized evanescent guided-wave SERS. The OCB provided qualitative insight into the orientation of the probe 4MPy and 4ABA molecules on the PNP substrate. The OCB was shown to resolve Fe^2+^ ion binding in water at the nanoscale by following changes in the SER spectra of 4MPy over time. A brief discussion of the plasmonic field-enhanced magneto-optic Kerr effect set the stage to explore how polarized evanescent wave SERS reveals vibrational information relevant to the 4ABA ligand interface between magnetite nanoparticles and surface-bound Au@Ag plasmonic nanoparticles. Ultra-high-resolution SEM images were used to map the locations of Au@Ag nanoparticles for FDTD simulations. The simulations confirmed the presence of localized “hot spots” involving closely space core–shell nanoparticles. These hot spots are possible contributors to the SER effect in the evanescent field.

Clearly, the merits of the OCB can be expanded in future work to a broader range of molecular probes and to include more complex ion binding events, including in situ fluid phase scattering to reveal binding kinetics, and the quantitative assessment of the performance of the OCB in relation to relevant and alternative evanescent wave devices. We note that the recent work of Torres-Torres et al. on hierarchical nanostructures for biosensing gives strong incentive to advance our research on the OCB in the direction of nanomedicine [54]. Our present work, nevertheless, contributes new knowledge to the optical sensing field by providing an integrated chip and Raman spectrograph system that combines polarization control and plasmonic excitation to enhance evanescent wave SERS. In this sense, the OCB can be viewed as complimentary, and even supplementary, to other techniques that probe interfaces at the nanoscale.

## Data Availability

The original contributions presented in the study are included in the article/Supplementary Materials, further inquiries can be directed to the corresponding author.

## Supplementary Materials

The supporting information document can be downloaded at: https://www.mdpi.com/article/10.3390/bios14090409/s1, Figure S1: Schematic of beam steering and prism coupling assemblies used to excite polarized evanescent guided wave SERS from a nanoplasmonic OCB. The photograph at the left is a top view of the OCB showing a guided wave. Raman is scattered orthogonal to the plane of the photograph through the collection optics and into the spectrograph (see side view image of OCB at the right hand side of the illustration); Figure S2: Top: High resolution SEM image showing sparsely distributed Au@Ag NPs on a section of the OCB. Bottom: For the simulations, interparticle distances were idealized by centering black discs over the irregularly shaped particles. These were then mapped onto the grid and yellow-bounded region of the slab waveguide in the (b) bottom image. The side length of each square of the grid is 50 nm. (b) Perspective of the OCB structure decorated with sparsely distributed Au@Ag core-shell nanoparticles mimicking that of the actual OCB calibrated from the SEM experiment; Figure S3: Ultra-high resolution SEM image of Au@Ag NPs immobilized on glass substrate using APTES; Figure S4: (A) High-resolution SEM image of 4ABA-functionalized MNPS deposited on Au@Ag PNPs immobilized on glass substrate using APTES; (B) XPS analysis of O1s and N1s spectra of MNPs prior to and after incubation in 4ABA solution; Figure S5: Raman spectra of crystalline 4ABA using either TE- or TM-polarized light. References [55,56,57,58,59,60,61] are cited in the Supplementary Materials.
