# Supplementary material for: Polarized and Evanescent Guided Wave Surface-Enhanced Raman Spectroscopy of Ligand Interactions on a Plasmonic Nanoparticle Optical Chemical Bench
Source: Biosensors (Basel). 2024 Aug 23;14(9):409. doi: 10.3390/bios14090409 (PMC11430309; doi:10.3390/bios14090409)
Supplement: Supplementary file 1 [file biosensors-14-00409-s001.zip › biosensors-3098123-supplementary.pdf]

Supporting Information

Experimental Procedure

Synthesis of Au@Ag NPs. Au@Ag NPs were prepared by a seed-mediated method adapted from the procedure of Calagua et al. [1]. A gold seed solution was prepared by dissolving 0.04g of $HAuCl_4 \cdot 3H_2O$ (Sigma-Aldrich) in 100 mL of Milli-Q water. The solution was constantly stirred and brought to a boil. A second solution of 0.114g of trisodium citrate dihydrate (Sigma-Aldrich) in 1 mL of Milli-Q water was prepared. The citrate solution was added to the $HAuCl_4$ solution in one batch. The resulting solution was boiled for an additional 15 min and then cooled to room temperature in an ice bath.

To grow the gold core, 0.002g of $HAuCl_4 \cdot 3H_2O$ was dissolved in 20 mL of Milli-Q water. A total of 20 µL of 1N HCl, and then 200 µL of the previously prepared gold seed solution were added. The mixture was vigorously stirred until it was homogeneous before 100 µL of a freshly prepared 0.1 M ascorbic acid solution (Sigma-Aldrich) was added. This was followed by the addition of 100 µL of 0.3 M sodium citrate.

To grow the silver shell, 200 µL of a 0.1 M $AgNO_3$ solution was added to the 20 mL of the gold core suspension prepared in the previous step. A second solution was prepared by diluting 100 µL of a 0.1 M ascorbic acid solution in 5 mL of Milli-Q water. This ascorbic acid solution was added dropwise to the gold core solution. The reaction was allowed to proceed for 20 minutes. The resulting Au@Ag NPs were used immediately.

Preparation of the OCB by functionalization with Au@Ag nanoparticles. All steps were carried out under anaerobic conditions with a Schlenk manifold. Multimode slab optical waveguides were prepared from 200 μm-thick HiQa™ glass coverslips (Sigma-Aldrich) cut to the appropriate size. The coverslips were cleaned with soapy water (Sparkleen™, Fisher Scientific) and ethanol before immersion in a piranha solution for 1 h (piranha solutions are strongly acidic and a strong oxidizer; the solution used consisted of 3 parts concentrated sulfuric acid and 1 part 30 wt. % hydrogen peroxide). The cleaned waveguide was rinsed with copious amount of deionized water until the pH of the rinse was neutral. The waveguide was then rinsed again with ethanol before being placed in a round-bottom Schlenk flask equipped with a magnetic stir bar. The flask was evacuated, and then backfilled with argon. Degassed anhydrous toluene (15 mL) and (3-aminopropyl)triethoxysilane (99%, Sigma-Aldrich, 0.5 mL) were injected by syringe into the flask. The reaction mixture was heated to 70 ℃ for 1 h under an argon atmosphere. Afterward, the system was cooled to room temperature, and the liquid reaction medium was removed via a syringe. Anhydrous toluene (Sigma-Aldrich) and then methanol (Sigma-Aldrich) were introduced via syringe to rinse the waveguide. The wash fluids were removed, and the system was evacuated to dryness. The functionalized waveguide was annealed under vacuum at 100 ℃ for 1 h before it was immersed in a suspension of freshly synthesized Au@Ag NPs to yield a porous mat of aggregated PNPs.

Binding of 4MPy or 4ABA molecules onto the OCB. The binding of 4MPy molecules was achieved by submerging the as-prepared Au@Ag coated glass waveguide into a 4MPy solution in ethanol (6×10^{-3}M, 4MPy, Sigma-Aldrich) for 30 min with occasional agitation.

Similarly, to bind 4ABA to the waveguide surface, the same procedure was followed with a 4ABA solution ethanol (6×10^{-3}M, 4ABA, Sigma-Aldrich).

Binding of Fe^{2+} to 4MPy on the OCB. A 0.5 mM Fe^{2+} solution was prepared by dissolving 152 mg of $FeSO_4$ in deionized water. A 4MPy-functionalized Au@Ag OCB was immersed in the Fe^{2+} solution and agitated lightly. Different samples were allowed to react for 0, 8, 20, 35, and 50 minutes.

Binding 4ABA-functionalized magnetite nanoparticles onto the OCB. Magnetite nanoparticles (MNPs) were synthesized using a modified coprecipitation protocol for magnetite nanoparticles [2]. Briefly, 1.5 g of $FeCl_2\cdot4H_2O$ (Sigma-Aldrich) and 4.1 g of $FeCl_3\cdot6H_2O$ (Sigma-Aldrich) were dissolved in 100 ml of vigorously stirred deionized water at 70 °C under a nitrogen gas flow. A 20 ml volume of 5 wt% NH_4OH was added dropwise until the solution color changed from orange to black. The product was collected by magnetic separation and washed with a copious amount of deionized water until the supernatant pH was neutral. Oxidation of the product was expected since no coating agent was added during synthesis.

MNPs were functionalized with 4ABA (Sigma-Aldrich) by incubating the MNPs in a 2×10^{-2} M 4ABA solution in 95% ethanol. The surface-modified MNPs were extracted using a magnet and re-dispersed in fresh 95% ethanol. A nanoplasmonic Au@Ag waveguide OCB was then dipped into the ABA-MNP suspension.

X-ray photoelectron spectroscopy (XPS). XPS was performed using a Thermo Scientific K-Alpha X-Ray Photoelectron Spectrometer. The X-ray source employed was an Al-Kα X-ray of 1486.6 eV. The X-ray beam was directed at an incident angle of 60° from normal (30° from the horizontal plane of the sample) with a focal spot size of 400 µm. A flood gun was used alongside the X-ray gun to reduce peak shifting. Samples were mounted onto conductive copper tape. The XPS vacuum chamber was maintained at $<2 \times 10^{-7}$ mbar throughout the experiments. N(1s) and O(1s) spectra were collected for the as-synthesized magnetite nanoparticles or after they were incubated in a 2×10^{-2} M 4ABA solution in 95% ethanol.

Integrated optics (IO) SERS spectrometry. A custom-built IO-SERS instrument was used to acquire polarization-dependent enhanced Raman scattering from the nanoplasmonic OCBs. A diagram of the scattering geometry is shown in **Figure S1**. A Laser Quantum Excel model 08542 diode laser provided 532 nm CW light. The 1.5 mm circular cross-section beam was directed by 45° $\lambda/2$ flatness mirrors to the entrance port of a precision rotation stage that supported a beam-steering apparatus. The beam steerer incorporated three 45° $\lambda/2$ flatness mirrors, a Glan–Thompson polarizer, a halfwave plate, and an achromatic plano-convex focusing lens. These components are rotated simultaneously about the axis of the entrance hole for the incident beam that enters the beam-steering unit parallel to the plane of the optical table. The slab waveguide OCB was mounted in a stainless-steel holder comprising thermally matched components. The holder was mounted on an xyz translation stage (Newport) bolted to the vibration-isolated optical table through a dynamically damped optical post. A guided wave was launched into the

waveguide OCB via prism coupling. Accordingly, the steered laser beam was focused onto the prism hypotenuse, which refracted the beam to the back bottom edge of the prism. The prism position was adjusted by an xyz translation stage to hunt for a guided wave. Light scattered from the waveguide streak was collected through optics that were f-number-matched to the f/6.4 550 mm focal length Triax spectrograph (Horiba). The optical train included a fast lens followed by collimating and focusing achromatic lenses to align the waveguide streak through a polarization scrambler and a holographic super notch filter (Kaiser Optics) onto the vertical slit of the spectrograph. With the polarizer in position in the beam-steering apparatus, a TE or TM wave polarization could be excited in the slab waveguide OCB. The laser power at the coupling prism was approximately 20 mW, except for the 4ABA@MNP@Au@Ag sample, which required ~ 50 mW to generate a sufficient Raman signal. The Horiba Triax 550 Raman spectrograph was equipped with a liquid nitrogen-cooled coupled device detector (CCD) camera. The Raman signal was dispersed over the CCD chip by a holographic grating (1800 grooves mm^{-1}). A 100 s acquisition time was typically employed for each 500 cm^{-1} scan window. Spectra from an average of 3 acquisitions were plotted.

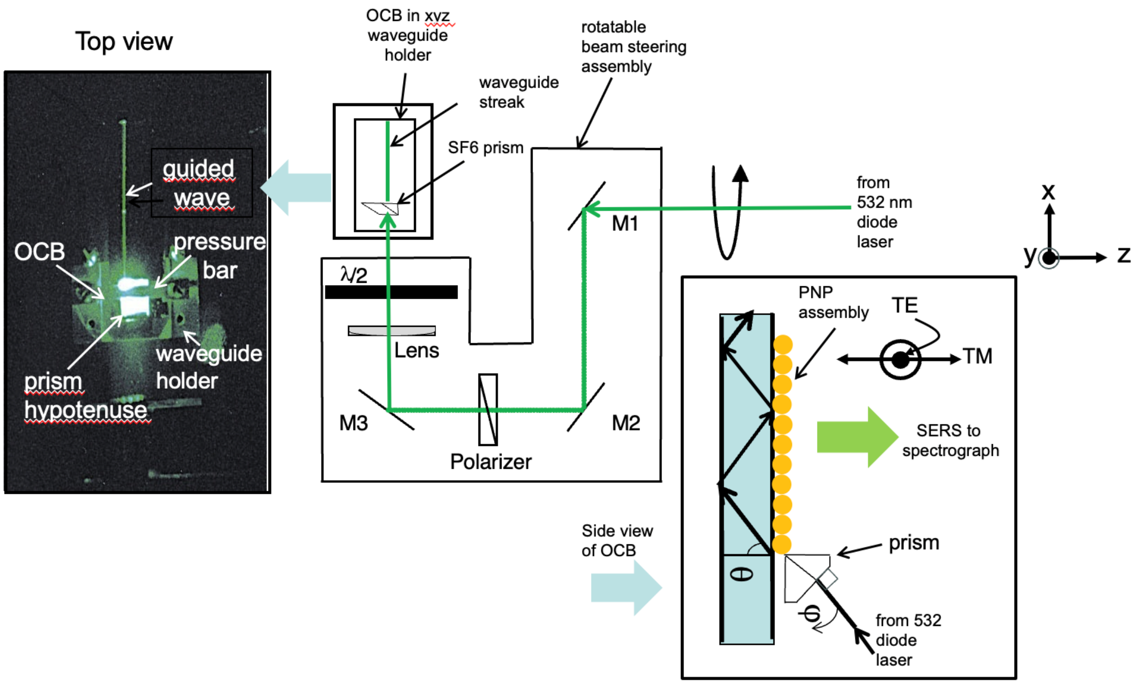

Figure S1. Schematic of beam-steering and prism-coupling assemblies used to excite polarized evanescent guided wave SER from a nanoplasmonic OCB. The photograph at the left is a top view of the OCB showing a guided wave. The Raman signal was scattered orthogonally to the plane of the photograph through the collection optics and into the spectrograph (see side view image of OCB at the right-hand side of the illustration).

Prism coupling was performed with a custom-designed SF6 prism from Precision Optical, USA. Its prism refractive index is 1.81 at 532 nm. A metal waveguide holder machined from thermally matched stainless-steel alloy held the prism and waveguide assembly in place. The width of the air gap between the prism base and the waveguide layer is critical for successful coupling. When coupling conditions are met, ripple-like "m-lines" (interference fringes) appear beneath the prism base lying parallel to the right-angle intersection of the two vertical planes. The prism position and gap distance were maintained by a top spring-loaded pressure bar, where the pressure was maintained by collections of Bell washers. The coupling angle was selected by varying the rotation angle

of the laser beam incident on the prism hypotenuse face with the beam point of focus at the prism bottom edge located on the rotation axis of the beam-steering arm. The waveguide holder was attached to an adjustable mount which allows for linear movement in the x, y, and z directions. When the correct prism gap and coupling angle are achieved, the incoming 532 nm laser ray excites a standing wave in the slab waveguide.

Scanning Electron Microscopy (SEM). High-resolution SEM imaging and corresponding energy-dispersive X-ray spectroscopy (EDX) were performed using a Helios NanoLab™ 660 instrument that combines field-emission SEM (FESEM) and focused ion beam (FIB) capability. An Everhart–Thornley Detector (ETD) and a high voltage of 10.0 kV were selected.

Transmission Electron Microscopy (TEM). Light-field and dark-field TEM, as well as the corresponding elemental mapping of the Au@Ag nanoparticles by energy-dispersive X-ray (EDX)analysis, were performed using an FEI Titan Krios 300 kV Cryo-STEM.

Simulation of plasmonic electric field. The resonant optical response of the Au@Ag nanoparticles at 532 nm was investigated using three-dimensional finite difference time domain (3D FDTD) simulations (Lumerical Solutions, Vancouver, BC, Canada). A miniature SiO_2 waveguide (1 μm in the x direction, 0.35 μm in the y direction, and 0.35 μm in the z thickness) was created to model the real-life waveguide. The z thickness cut to 0.35 μm provided a view of the upper region of the multimode slab waveguide where the

evanescent field interacts with the plasmonic nanoparticles. The boundary conditions were PMLs (Perfectly Matched Layers) in the y and z directions, which absorb light with minimal reflections to respect the waveguide boundary, and periodic in the x direction to maintain continuity. Au@Ag core–shell nanoparticles with a silver shell radius of 25 nm and a gold core radius of 10 nm were introduced as an idealized representation of the Au@Ag core–shell nanoparticles. Calculations were performed within the Mie theory in the quasistatic dipole approximation using the bulk dielectric functions of the metallic core and shell without mean free path corrections to account for size confinement effects. The core–shell nanoparticles were placed sparsely on the waveguide by mapping the approximate size and locations of the nanoparticles, as observed by high-resolution SEM on a randomly selected section of the real-life waveguide (Figure S2 a, b, bottom). A pulse of light at 532 ± 10 nm was launched through the center of the waveguide with either TE or TM polarization. The electric field distribution of a selected area on the waveguide model was simulated using a 1 nm mesh size (Figure S2 b). The yellow box in the xy plane indicates where the electric field intensity profiles were calculated and plotted. The wave propagation occurred in the x direction according to the laboratory coordinate system.

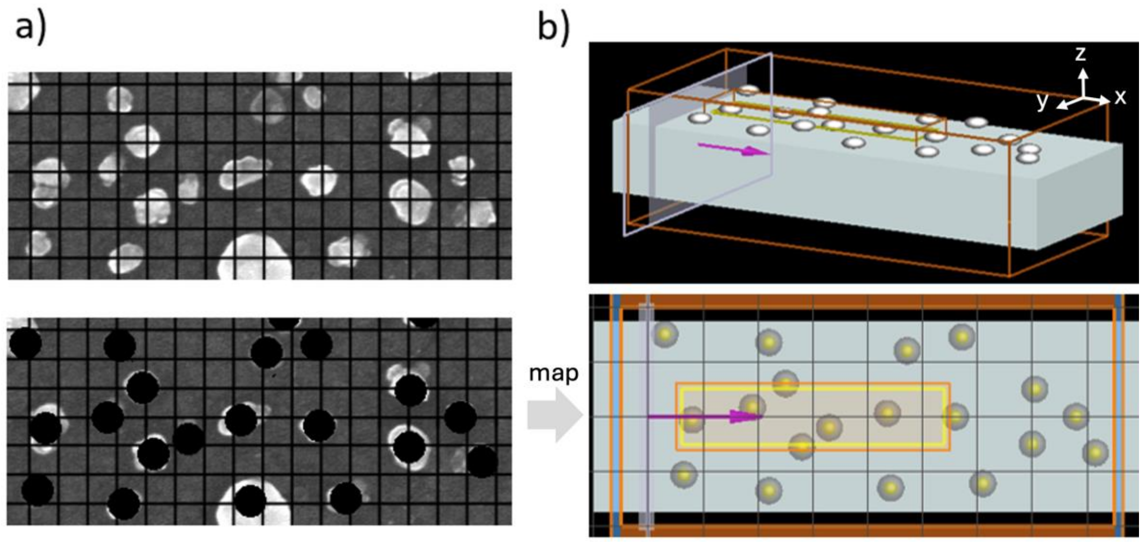

Figure S2. (**a**) Top: High-resolution SEM image showing sparsely distributed Au@Ag NPs on a section of the OCB. Bottom: For the simulations, interparticle distances were idealized by centering black discs over the irregularly shaped particles. These were then mapped onto the grid and yellow-bounded region of the slab waveguide in the bottom image in (b). The side length of each square of the grid is 50 nm. (**b**) Perspective of the OCB structure decorated with sparsely distributed Au@Ag core–shell nanoparticles mimicking that of the actual OCB calibrated from the SEM experiment.

Figure S3 shows an ultra-high-resolution SEM image of the OCB waveguide featuring bimetallic Au@Ag NPs anchored by aminopropyl groups grafted to the glass substrate. Individual nanoparticles were dotted over the surface, which showed evidence of aggregation among the particles. Overall, the waveguide surface was sparsely covered with Au@Ag NPs. Immobilization of the Au@Ag NPs on the glass substrate led to further broadening of the plasmon absorption band (Figure 3c in the text). These effects likely

resulted from the clustering of NPs on the substrate, mutual polarization among closely associated NPs, and multiple scattering from large aggregates [3, 4].

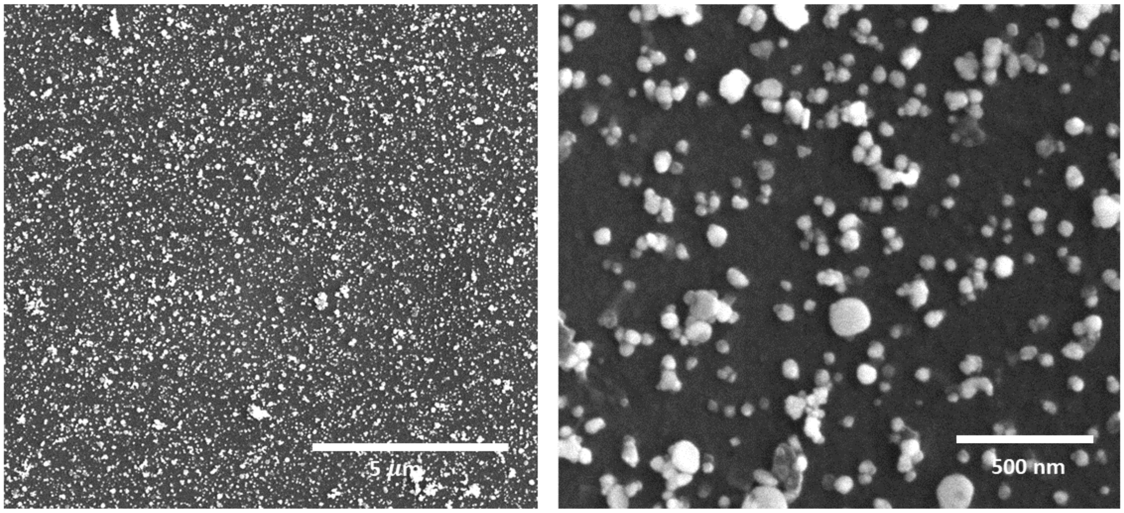

Figure S3. Ultra-high-resolution SEM image of Au@Ag NPs immobilized on glass substrate using APTES.

4ABA-functionalized MNPs deposited on plasmonic Au@Ag NPs on the OCB

High-resolution SEM images of MNPs deposited on the Au@Ag NP-covered waveguide is shown in Figure S4A.

XPS was used to examine the features of the binding of 4ABA onto the MNPs (Figure S4B). Comparing the O1s spectra from pre-functionalized MNPs and after MNPs incubation in the 4ABA solution, a significant increase in O-C species (531.3 eV) was observed. This difference indicates the presence of COO^- species capable of monodentate or bidentate binding to the MNP surface through electrostatic or chemisorption [5]. Although the electron densities on the oxygen atoms were nonequivalent in the different

carboxylate binding modes, XPS could not resolve the peaks [5]. The N1s spectra centered at 399.7 eV revealed an abundance of $-NH_2$ group on the surface of the MNPs after incubation in the 4ABA solution. Prior to incubation, only a small amount of N-H was detected, likely remnants from the coprecipitation synthesis of the MNPs, which employed ammonium hydroxide as a base. A small peak at 398.3 eV was also detected in the post-incubation N1s spectrum. It was assigned to N-Fe, suggesting possible covalent bonding between nitrogen and iron [6, 7]. According to the XPS results, the NH_2 and COO^- groups were bound to the surface of the MNPs.

(A)

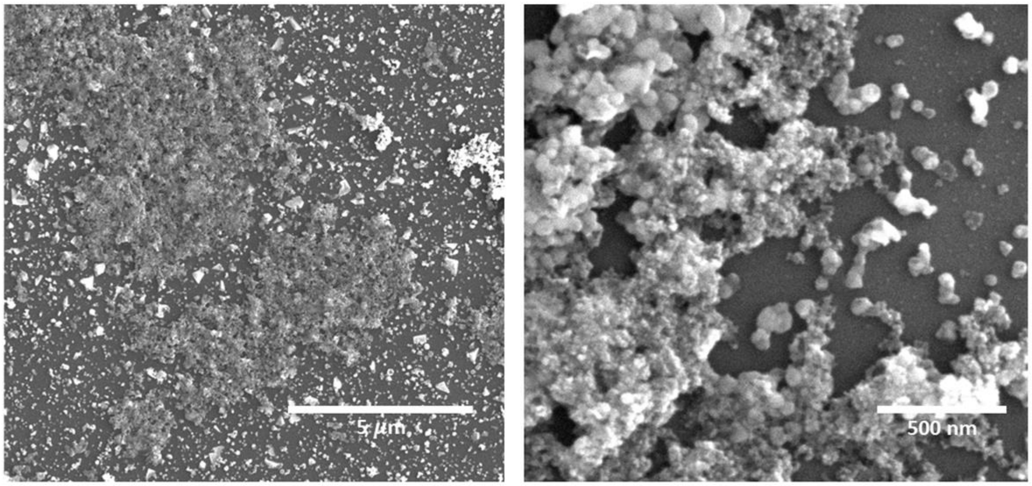

(B)

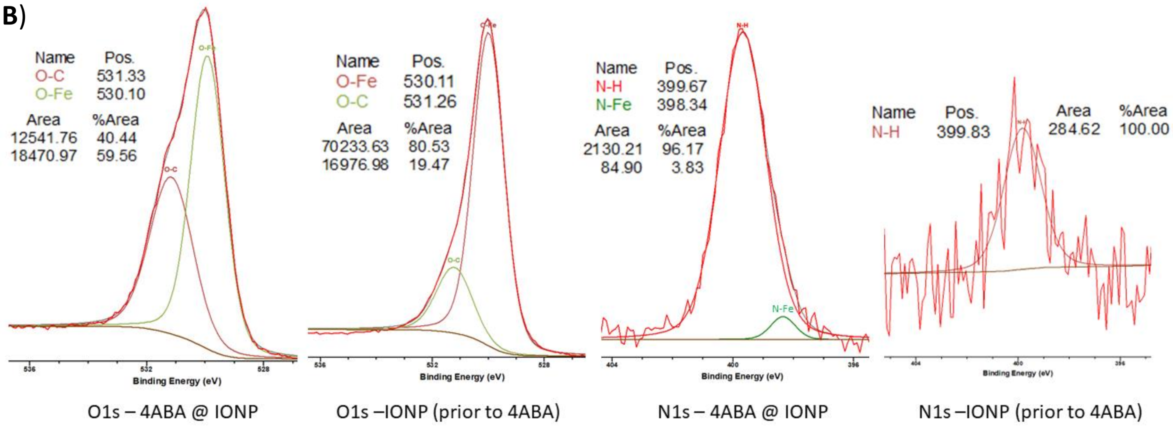

Figure S4. (**A**) Ultra-high-resolution SEM images of 4ABA-functionalized MNPS deposited on Au@Ag NPs immobilized on the OCB by APTES; (**B**) XPS fittings of O1s and N1s spectra of MNPs prior to and after incubation in 4ABA solution.

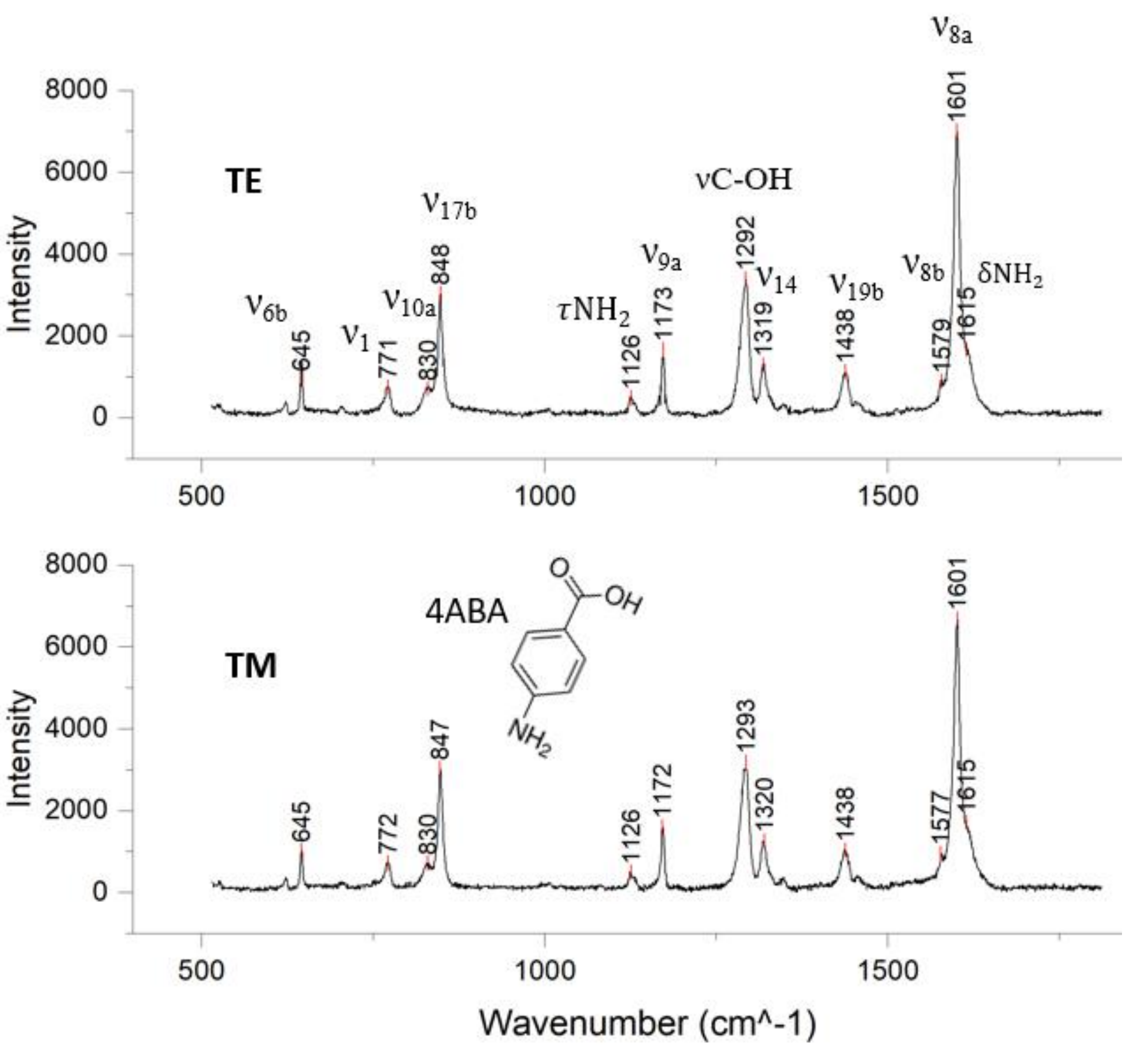

Figure S5. Raman spectra of polycrystalline powder of 4ABA using either TE- or TM-polarized light.

References

1. Calagua, A., Alarcon, H., Paraguay, F. and Rodriguez, J., 2015. Synthesis and characterization of bimetallic gold-silver core-shell nanoparticles: a green approach. *Advances in Nanoparticles*, *4*(04), p.116.
2. Petcharoen, K. and Sirivat, A., 2012. Synthesis and characterization of magnetite nanoparticles via the chemical coprecipitation method. *Materials Science and Engineering: B*, *177*(5), pp.421-427.
3. Ghosh, S.K. and Pal, T., 2007. Interparticle coupling effect on the surface plasmon resonance of gold nanoparticles: from theory to applications. *Chemical Reviews*, *107*(11), pp.4797-4862.
4. He, R.X., Liang, R., Peng, P. and Zhou, Y.N., 2017. Effect of the size of silver nanoparticles on SERS signal enhancement. *Journal of Nanoparticle Research*, *19*(8), pp.1-10.
5. Wilson, D. and Langell, M.A., 2014. XPS analysis of oleylamine/oleic acid capped Fe3O4 nanoparticles as a function of temperature. *Applied Surface Science*, *303*, pp.6-13.
6. Wagner, A.J., Wolfe, G.M. and Fairbrother, D.H., 2003. Reactivity of vapor-deposited metal atoms with nitrogen-containing polymers and organic surfaces studied by in situ XPS. *Applied Surface Science*, *219*(3-4), pp.317-328.
7. Degaga, G.D., Trought, M., Nemsak, S., Crumlin, E.J., Seel, M., Pandey, R. and Perrine, K.A., 2020. Investigation of N_2 adsorption on Fe_3O_4 (001) using ambient pressure X-ray photoelectron spectroscopy and density functional theory. *The Journal of Chemical Physics*, *152*(5), p.054717.
